# Cytochrome P450 CYP2E1 Suppression Ameliorates Cerebral Ischemia Reperfusion Injury

**DOI:** 10.3390/antiox10010052

**Published:** 2021-01-05

**Authors:** Jin Yu, Hong Zhu, Mark S. Kindy, Saeid Taheri

**Affiliations:** 1 Department of Pharmaceutical Sciences, College of Pharmacy, University of South Florida, 12901 Bruce B. Downs Blvd., MDC 30, Tampa, FL 33612, USA; jiny@usf.edu (J.Y.); Hongz@usf.edu (H.Z.); kindym@usf.edu (M.S.K.); 2 James A. Haley Veterans Affairs Medical Center, Tampa, FL 33612, USA; 3 USF Heart Institute, Tampa, FL 33602, USA

**Keywords:** blood-brain barrier, CYP2E1, inflammation, ischemia/reperfusion, oxidative stress

## Abstract

Despite existing strong evidence on oxidative markers overproduction following ischemia/reperfusion (I/R), the mechanism by which oxidative enzyme Cytochrome P450-2E1 (CYP2E1) contributes to I/R outcomes is not clear. In this study, we sought to evaluate the functional significance of CYP2E1 in I/R. CYP2E1 KO mice and controls were subjected to middle cerebral artery occlusion (MCAo-90 min) followed by 24 h of reperfusion to induce focal I/R injury as an acute stage model. Then, histological and chemical analyses were conducted to investigate the role of CYP2E1 in lesion volume, oxidative stress, and inflammation exacerbation. Furthermore, the role of CYP2E1 on the blood-brain barrier (BBB) integrity was investigated by measuring 20-hydroxyecosatetraenoic acid (20-HETE) activity, as well as, in vivo BBB transfer rate. Following I/R, the CYP2E1 KO mice exhibited a significantly lower lesion volume, and neurological deficits compared to controls (*p* < 0.005). Moreover, reactive oxygen species (ROS) production, apoptosis, and neurodegeneration were significantly lower in the CYP2E1(−/−) I/R group (*p* < 0.001). The BBB damage was significantly lower in CYP2E1(−/−) mice compared to wild-type (WT) (*p* < 0.001), while 20-HETE production was increased by 41%. Besides, inflammatory cytokines expression and the number of activated microglia were significantly lower in CYP2E1(−/−) mice following I/R. CYP2E1 suppression ameliorates I/R injury and protects BBB integrity by reducing both oxidative stress and inflammation.

## 1. Introduction

Clinically effective treatments to reduce ischemia-reperfusion (I/R) injury are still a major unfulfilled medical need. Several barriers and limitations exist in the wide use of favorite reperfusion methods. For example, reperfusion of ischemic cells results in a reactive oxygen species (ROS) burst that contributes to lipid peroxidation, as well as DNA oxidation and irreversible tissue injury. The most important sources of ROS are mitochondrial dysfunction, NADPH oxidases, cellular enzymes, as well as metabolic enzymes such as the cytochrome P450 family [1]. Cytochrome P450-2E1 (CYP2E1), a subfamily of cytochrome P450, is one of the most active Cytochrome in ROS production [2,3], and inflammation [4]. Its induction has been associated with ROS production in the liver [5].

While CYP2E1 is predominantly expressed in the liver, significant levels of CYP2E1 are expressed and induced in many extrahepatic tissues (e.g., lung, kidney, and the brain) [6,7,8]. Compared to other P450 enzymes, a significant amount of CYP2E1 is expressed in the human brain [6,8,9], as well as the rodent brain [7]. Specifically, Howard et al. have shown that in untreated rodents, low quantities of CYP2E1 were present predominantly in evolutionarily older areas of the allocortex, such as the olfactory bulbs, olfactory cortex, hippocampus, cerebellum, and brainstem [8]. CYP2E1 is endogenously expressed in primary cultures of cerebellar granule neurons [10]. It is also highly inducible in astrocytes following ischemic or mechanical damage [11,12]. CYP2E1 has been found in different cell compartments such as mitochondria, plasma membrane, Golgi apparatus, and the endoplasmic reticulum (for review, see e.g., [13]).

Brain indigenous substrates of CYP2E1 are arachidonic acids, linoleic acids, and oleic acids gluconeogenic precursors and estrogenic metabolites [14]. Metabolites of CYP2E1 are18 and 19-hydroxyecosatetraenoic acid 18-, 19-HETE, hydroxylinoleic acid, and 17-, 18-hydroxyoleic acids that play a vital role in membranes’ structure and function [15,16]. However, it has been shown that reduced CYP2E1 expression and superoxide formation enhances 20-hydroxyecosatetraenoic acid (20-HETE) levels [17]. Using a rat model of ischemia, studies have shown that 20-HETE inhibition reduces infarct and improves cerebral blood flow (CBF) [18]. Moreover, in human neurons, CYP2E1 is known to generate ROS and nitric oxide through the induction of NADPH/xanthine oxidase and nitric oxide synthase [19]. However, a clear connection between I/R insult and cascade of cerebral tissue damage because of CYP2E1 is missing, moreover, it is not known how the CYP2E1 activity impacts the blood-brain barrier (BBB) function if the aging exacerbates oxidative damage via increasing CYP2E1 activity. Therefore, it is important to provide knowledge on how oxidative stress exacerbates by age and how its accumulation plays important role in complications following I/R.

Cerebral ischemia is shown to induce CYP2E1 expression [20]. Increased CYP2E1 expression has been connected to hypoxia and inflammation [21,22]. Though studies imply a vital role for CYP2E1 in oxidative stress and inflammation following ischemia, the most important questions on how CYP2E1 activity exacerbates I/R injury remain unanswered. In the present study, we have therefore investigated the significance of CYP2E1 in cerebral damage, the extent of oxidative stress, and inflammatory markers activity following I/R. In addition, we have investigated the CYP2E1 role in 20-HETE expression and especially on BBB impairment following I/R.

## 2. Materials and Methods

### 2.1. Subjects, Legal Issues, Randomization, and Statistical Planning

CYP2E1(−/−) mice (CYP2E1-null) on the C57BL/6 background selected from our breeding colony established at The University of South Florida (breeder on the SV/129 background was kindly provided by Dr. Frank J. Gonzalez; Laboratory of Metabolism, National Cancer Institute, Bethesda, MD USA then converted to C57BL/6 background to ensure consistency in comparing our data with others) [2] for this experiment (M/F, 12–18 w, 25–30 g). The clone of mice maintained by breeding CYP2E1(−/−) males with CYP2E1(−/−) females. A complete absence of CYP2E1 protein and mRNA was confirmed by immunoblotting and northern blotting. C57BL/6 wild-type (WT) mice were purchased from The Jackson Laboratory (Bar Harbor, ME, USA). All mice housed in temperature-controlled animal facilities with 12 h light/12 h dark cycles and permitted consumption of tap water and Purina standard chow ad libitum. All animal procedures were conducted in accordance with the “Guide for the Care and Use of Laboratory Animals” (Institute of Laboratory Animal Resources on Life Sciences, National Research Council, 1996) and approval by USF IACUC #7127R. We also followed the Stroke Therapy Academic Industry Roundtable (STAIR) recommendations [23]. Experiments were strictly randomized and blinded. Statistical planning assumed an α-error of 5% and a β-error of 20%. The data that support the findings of this study are available from the corresponding author upon reasonable request.

### 2.2. Experimental Groups

Three experimental groups CYP2E1 KO (*n* = 12F + 12M), WT (*n* = 12F + 12M), and Sham groups (*n* = 5M + 5F), unless otherwise mentioned, for both sexes were studied for (1) The role of CYP2E1 on ROS activity, (2) Stroke complication because of CYP2E1 expression (permanent vs. transient), and (3) The role of CYP2E1 in neurovascular inflammation, 20-HETE expression, and the BBB health. Animals were randomly assigned to the experimental groups.

### 2.3. Cerebral Ischemia/Reperfusion Model

The mice were subjected to 90 min middle cerebral artery occlusion (MCAo) to induce permanent MCAo or followed by 24 h reperfusion to induce focal cerebral I/R injury or transient MCAo (tMCAo). The filament occlusion was omitted to induce sham-operated models. To induce MCAo, mice surgically manipulated to expose the common carotid arteries. A silicon-coated filament inserted into the left common carotid artery, up through the internal carotid artery to block the MCA. For the permanent MCAo model, the filament remained in place permanently. But for tMCAo the filament remained in place for 90 min (occlusion) and then retracted to allow for cerebral region reperfusion. During the surgery, 2.0% isoflurane was used for induction and 1.0–2.0% for maintenance. As for post-surgery treatments, mice were given the same amount of analgesic agents and normal saline 2 h after the operation. Rules for inclusions and exclusions of the models are presented in Materials and Methods in the Data Supplement.

### 2.4. Neurological Deficit Assessments

The neurological deficits were assessed and scored on a 5-point scale based on the report of Longa et al. [24]. Mouse with no neurological deficit scored 0. Left forepaws with flexion, adduction, and failure to extend fully scored 1. Circulating and rotating to the left when crawling scored 2. Mouse falling to the left scored 3. Not walking spontaneously scored 4, and dead mouse scored 5.

### 2.5. Assessment of Infarct Size by 2,3,5-Triphenyltetrazolium Chloride (TTC) Staining

After the reperfusion, five mice were randomly selected as samples from each group, the brains were removed and sectioned into five coronal sections, 2 mm thick for TTC staining. Details on TTC staining and infarct volume measurement are presented in Materials and Methods in the Data Supplement.

### 2.6. Preparation of Brain Membranes

Total cell membranes were prepared because it has been shown that brain CYPs are present more in multiple membrane fractions, including microsomal, mitochondrial, and nuclear membranes [25,26,27]. The whole brain was used to prepare and extract brain membranes for further analysis. The protocol for brain membrane preparation is presented in Materials and Methods in the Data Supplement.

### 2.7. Screening CYP2E1 Enzyme Activity

CYP2E1 activity was assessed by measuring the rate of p-nitrophenol oxidation to p-nitrocatechol with samples of brain membranes as described previously [28,29,30,31]. Samples from the whole brain membranes were used to screen CYP2E1 activity. Details on the CYP2E1 activity measurement protocol are presented in Materials and Methods in the Data Supplement. 

### 2.8. Terminal Deoxynucleotidyl Transferase-Mediated dUTP-Biotin Nick End Labeling (TUNEL) Assay

TUNEL assay was used to assess neuronal apoptosis in the ischemic hemisphere. Coronal brain slices that contain the ischemic region and the corresponding brain regions in controls were used for the TUNEL assay. Immunohistochemistry protocols and antibodies are presented in Materials and Methods in the Data Supplement. 

### 2.9. Analysis of Inflammation

Tissue samples were taken from the ischemic region of the brain for microglial activity and astrogliosis analysis. Cerebral ionized calcium-binding adaptor protein-1 (Iba-1), glial fibrillary acidic protein (GFAP), and β-actin protein levels were determined at 24 h after I/R because Iba-1 and GFAP protein levels are known to be significantly increased at that time post-ischemia [32]. Immunohistochemistry protocols and antibodies are presented in Materials and Methods in the Data Supplement. 

### 2.10. Enzyme-Linked Immunosorbent Essay Analysis (ELISA)

Protein levels of inflammatory markers; tumor necrosis factor (TNF)-α, interleukin (IL)-6, and monocyte chemoattractant protein 1 (MCP-1) in the brain were measured using an ELISA kit (KMC0061c, BMS607-21NST, BMS6005 respectively, Thermo Fisher Scientific, San Jose, CA, USA) according to the manufacturer’s protocol. Tissue samples were taken from ischemic regions and corresponding regions in control brains. Samples were measured in duplicates. Readings from each sample were normalized for protein concentration. Moreover, tissue 20-HETE levels were assessed using 20-HETE ELISA kits (50-753-4354 R&D Inc, Detroit, MI, USA) according to the manufacturer’s instructions, and results were normalized to dry weight.

### 2.11. Analysis of Oxidative Stress

ROS generation was measured using the CM-H2DCFDA (Thermo Fisher Scientific, San Jose, CA, USA) a peroxide-sensitive fluorescent probe as described previously with minor modifications [33]. The homogenates from the whole brain were used to analyze the ROS generation. The details on the method are presented in Materials and Methods in the Data Supplement.

### 2.12. NeuroImaging

Magnetic resonance imaging was performed with 7T BioSpec MR Scanner (Bruker Biospin, Ettlingen, Germany) equipped with 500 mT/m gradient (rise time 80–120 μs), and a cryogenic quadrature RF surface coil (Bruker Biospin) as the RF transmitter/receiver [34] at 24 h of reperfusion. This imaging time was selected because of its importance as one of the milestones for ischemic evolution [35,36,37]. Animal imaging conditions and MR protocols details are presented in Materials and Methods in the Data Supplement.

### 2.13. Statistical Analysis

We conducted all statistical analyses in the “R” environment (R core team 2017). Study groups were blinded to the person (S.T.) who conducted the statistical analysis. Data are expressed as mean ± standard deviation (SD), and n refers to the number of animals used. All data sets were tested for normality using the Shapiro–Wilk test, and a subsequent unpaired *t*-test or Mann–Whitney test was applied based on parametric or non-parametric distribution, respectively. One-way analysis of variance (ANOVA) with Dunnett’s post-hoc was used for comparison of more than two data sets. Differences were considered to be significant at *p* < 0.05.

## 3. Results

### 3.1. Infarct Volume and Neurological Outcomes

**Figure 1:**
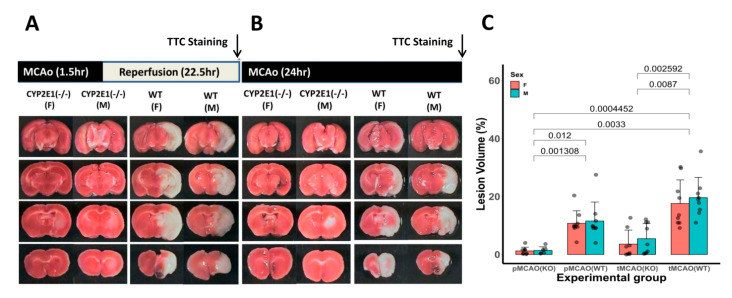
Genetic deletion of cytochrome P450-2E1 (CYP2E1) protects against cerebral ischemia/reperfusion (I/R) injury. Representative photomicrographs sets of 2,3,5-triphenyltetrazolium chloride (TTC)-stained brain slices for transient middle cerebral artery occlusion (MCAo) (tMCAo) and permanent MCAo at 24 h are shown in panels (**A**,**B**), respectively. Quantitative analysis of total brain infarct volume ratio (in %) is shown in panel (**C**). Male (M) and female (F) tMCAo (90 min) followed by 22.5 h of reperfusion and permanent ischemia along with aged-match controls were studied separately for lesion volume by TTC staining at 24 h of ischemic insult. Inhibition of CYP2E1 enzyme significantly decreased infarct volume in comparison with wild-type (WT) male. Values are presented as mean ± SD (*n* = 8–12 per group).

CYP2E1 inhibition significantly protected the brain from both MCAo (** *p* < 0.005), and tMCAo (* *p* < 0.01) as shown in a representative TTC stains and quantitative analysis of total brain infarct volume ratio. Though the lesion volume was higher in tMCAo (both genders) compared to the MCAo group, we did not find any statistically significant differences (Figure 1). We did not find any gender differences between lesion volume in both the MCAo model and tMCAo model. Remarkably, CYP2E1(−/−) mice showed significantly less severe clinical signs of I/R compared to WT mice, particularly the permanent ischemic group (1 ± 0.5 vs. 3 ± 0.5). See Figures S1 and S2 in Results in Data Supplement.

### 3.2. Expression of CYP2E1 Following I/R Insult

**Figure 2:**
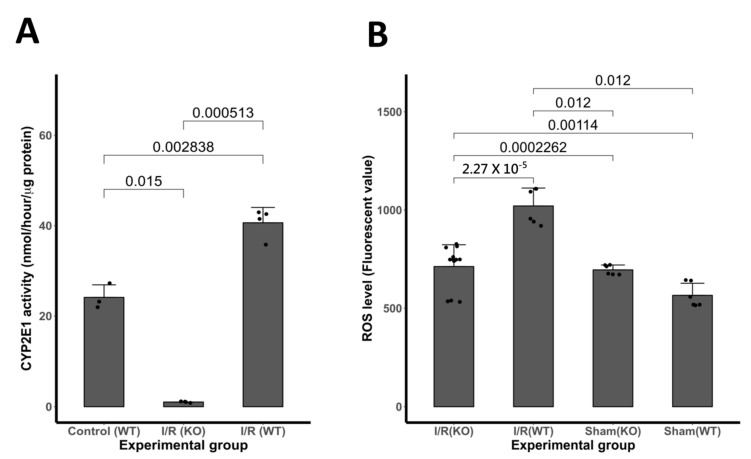
(**A**) CYP2E1 activity increases following I/R. Mice transient MCAo (tMCAo; 90 min occlusion and 24 h of reperfusion) models are compared with controls for cerebral CYP2E1 activity at 24 h. CYP2E1 activity was assessed by the nitrocatechol method [28]. CYP2E1 activity was increased significantly (61%) following transient MCAo compared with age- and sex-matched controls (*n* = 7). No significant CYP2E1 activity was found in the CYP2E1(−/−) group. (**B**). Ninety minutes I/R increases Reactive oxygen species (ROS) production in the brain, as determined by using chloromethyl derivative of dichlorofluorescein diacetate (MC-H_2_DCFDA) at 24 h post-reperfusion. The fluorescent signals generated with MC-H_2_DCFDA in the brain were increased with the insult of I/R. The analysis of fluorescence intensity illustrates the significant reduction in ROS production in CYP2E1(−/−) mouse after the insult of I/R in comparison to age-matched WT mice. No fluorescence was detected in wells without the incubation of MC-H_2_DCFDA. Data are shown as mean + SD. *n = 6* male per group.

We evaluated whether brain CYP2E1 activity elevates following I/R. We observed that CYP2E1 activity significantly increased (61%) following I/R in WT mice compared with age- and sex-matched controls (** *p* < 0.005, *n* = 8). As expected, no significant CYP2E1 activity found in CYP2E1(−/−) group (Figure 2A).

### 3.3. Expression of ROS Activity Following I/R Insult

Compared with WT controls, the ROS level was decreased by 1.5 fold (*p* < 0.001) in CYP2E1 KO. The analysis of fluorescence intensity illustrates the significant reduction in ROS production in CYP2E1 KO mice after the insult of I/R in comparison to age-matched WT mice. No fluorescence was detected in wells without the incubation of MC-H_2_DCFDA. Data are shown as mean + SD. *n =* 7 M per group, and *** *p* < 0.001 (Figure 2B).

### 3.4. Cytoprotective Effects of CYP2E1 Inhibition and Glial Activation

**Figure 3:**
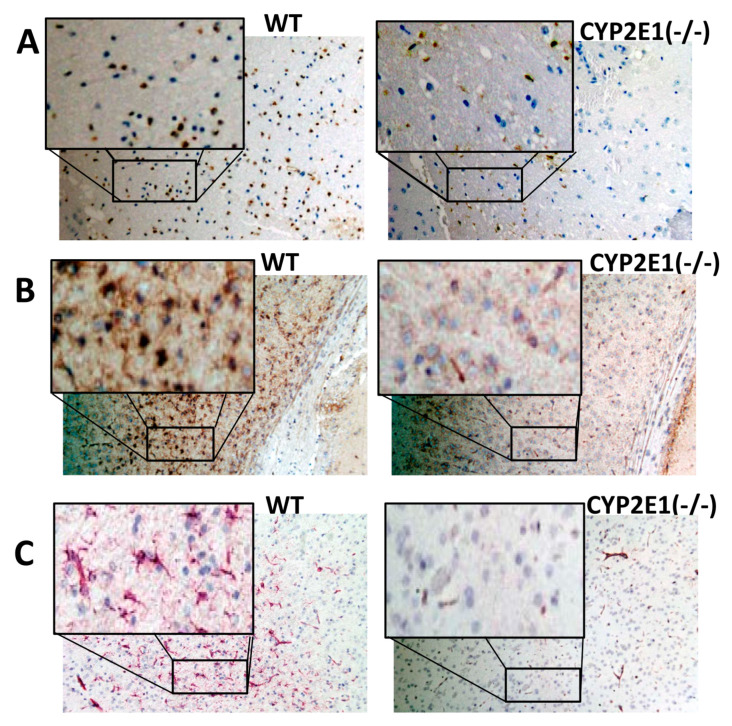
Apoptosis and neurodegeneration were reduced in CYP2E1(−/−) mice following I/R (tMCAo). (**A**) apoptosis of mice MCAo brain tissue in each group measured by terminal deoxynucleotidyl transferase-mediated dUTP-biotin nick end labeling (TUNEL) (magnification, ×200) in the MCA territory of the cortex. TUNEL-positive cells (brown staining) significantly decreased in CYP2E1 compared with those in the WT group. TUNEL assay was performed in the cortical cerebral sections. TUNEL-positive cells were counted. Representative figures show CYP2E1 inhibition reduces expression of (**B**) microglia/macrophage activation (ionized calcium-binding adaptor protein-1 (Iba-1) positive cells), and (**C**) astrogliosis following tMCAo and 24 h reperfusion (astrocytes glial fibrillary acidic protein (GFAP) positive cells) in the peri-infarct cortex. Astrocytes stained brown.

Apoptosis and neurodegeneration were reduced in CYP2E1(−/−) mice following tMCAo. Apoptosis of mice MCAo brain tissue in each group measured by TUNEL (magnification, ×200) in the middle cerebral artery territory of the cortex. TUNEL-positive cells (brown staining) significantly decreased in CYP2E1 compared with those in the WT group, see Figure 3A. CYP2E1 inhibition reduces expression of microglia/macrophage activation (Iba-1 positive cells-Figure 3B). Astrogliosis observed following tMCAo and 24 h reperfusion (GFAP positive cells) in the peri-infarct cortex. Activation of astrocytes (GFAP) was reduced in CYP2E1(−/−) mice (astrocytes stained brown in Figure 3C).

**Figure 4:**
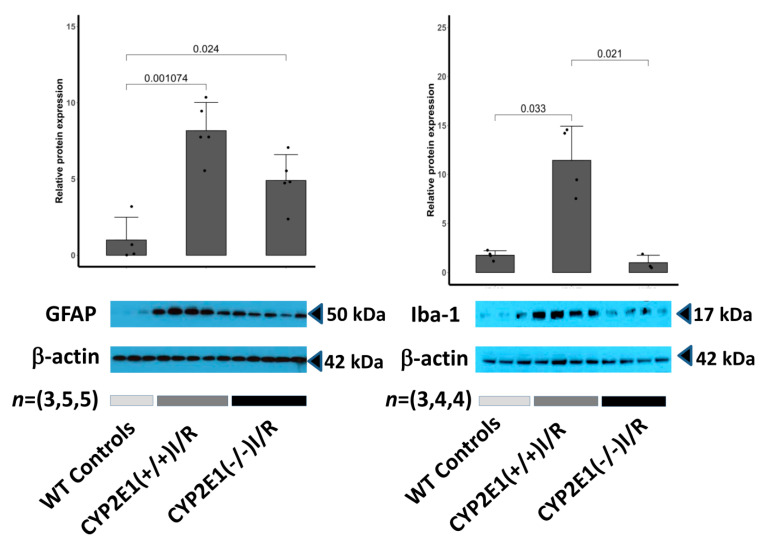
The quantified level of protein expression of Iba-1 (**right**) and GFAP (**left**) in the peri-infarct region of CYP2E1(−/−), CYP2E1(+/+), and control mice brains subject to 90 min MCAo reperfusion are shown in the (**top**) of the panel. Detection of the Iba-1 and GFAP in mouse peri-infarct areas CYP2E1 and deficient in CYP2E1 (knock-out; KO), was done by Western blot analysis using rabbit polyclonal anti-Iba-1 and anti-GFAP antibody. Data are represented as mean + SD (fold change relative to mean control value), *n* = (3,5,5) males per group (control, CYP2E1(+/+), and CYP2E1(−/−) for GFAP analysis and *n* = (3,4,4) males per group for Iba-1 analysis. A statistically significant difference between the expression of both GFAP and Iba-1 protein in CYP2E1(+/+) I/R and CYP2E1(−/−) I/R is observed. Representative Western blots of Iba-1 and GFAP protein levels in the brain of CYP2E1(−/−) I/R, CYP2E1(+/+) I/R, and WT control mice subject to 90 min MCAo are shown at the (**bottom**) of the panel. β-actin was used as a control for loading.

The quantified level of protein expression of Iba-1 (Right) and GFAP (Left) in the peri-infarct region of CYP2E1(−/−), CYP2E1(+/+), and WT control mice brains subject to 90 min I/R are shown in the top panel of Figure 4. A statistically significant difference between the expression of both GFAP and Iba-1 protein in CYP2E1(+/+) and CYP2E1(−/−) is observed (** *p* < 0.005 and *** *p* < 0.001, respectively). Representative Western blots of Iba-1 and GFAP protein levels in the ischemic brain are shown at the bottom of the panels respectively. β-actin was used as a control for loading.

### 3.5. Inflammatory Cytokine Protein Expression Was Reduced in CYP2E1(−/−) Mice Following tMCAo

**Figure 5:**
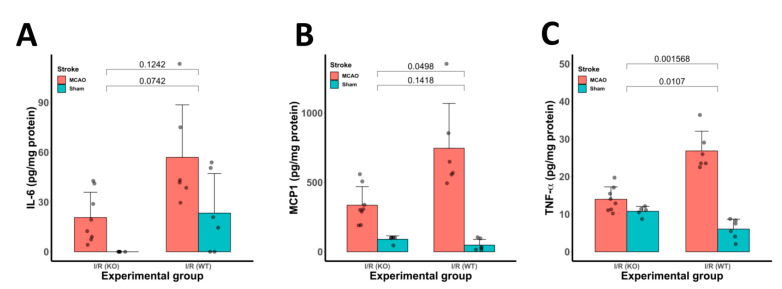
Inflammatory cytokine protein expression was reduced in CYP2E1(−/−) mice following I/R (tMCAo) insult. Bar graphs show the protein concentration of (**A**) interleukin (IL)-6, (**B**) monocyte chemoattractant protein 1 (MCP-1), and (**C**) tumor necrosis factor (TNF)-α in brain tissues at 24 h of MCAo, measured by ELISA. CYP2E1(−/−) groups show an almost two-fold reduction in inflammatory marker expression following I/R compared to WT I/R. Sham-operated animals did not show a marked increase in IL-6. The levels of brain IL-6, MCP1, and TNF-α were determined by ELISA kits. Data are presented as mean + SD, and *n* = 6–12.

We observed more than two-fold reduction in protein concentration of (A) IL-6 (*p* < 0.05), (B) MCP-1 (*p* < 0.05), and (C) TNF-α (*p* < 0.0001) in cerebral ischemic tissues at 24 h of I/R, as measured by ELISA. Data are presented as mean ± SD in Figure 5. Sham-operated controls did not show any significant increase in protein levels of assessed inflammatory markers.

### 3.6. Blood-Brain Barrier Leakage

**Figure 6:**
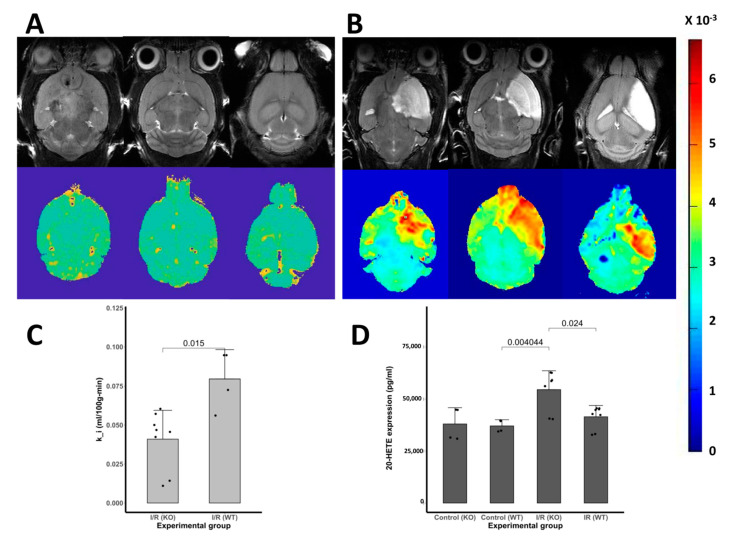
Ischemia/reperfusion triggers an increase in 20-hydroxyecosatetraenoic acid (20-HETE) synthesis and breakdown of the blood-brain barrier (BBB) in the brain of mice. (**A**) Representative BBB transfer rate map after I/R in CYP2E1(−/−) and (**B**) in WT control mice, in vivo MR quantification. Representative structural MRI (above) and corresponding quantitative BBB transfer rate map (below) are shown for three consecutive slices covering most of the ischemic lesion for both CYP2E1(−/−) and WT MCAo. Panel (**C**) represents a statistical comparison of BBB transfer rates between CYP2E1(−/−) MCAo and WT MCAo as measured by MRI. Furthermore, 20-HETE synthesis following the insult of I/R was increased significantly (*p* < 0.05) in CYP2E1(−/−) mice (36% of control). However, the 20-HETE synthesis increment in WT- I/R was not statistically significant compare to WT controls (12% of WT control). Data are presented as mean ± SD in panel (**D**). *n* = 8–10. Mouse MCAo was subjected to 90 min MCA occlusion and 24 h of reperfusion. In this CYP2E1 (−/−) model, in comparison to WT controls, lesion volume was significantly lower. Interestingly, there was no significant damage to BBB as measured by the rate of leakage of the contrast agent (Gd-DTPA) from the blood into cerebral tissues through BBB. Anatomical images acquired with RARE (Rapid Acquisition with Relaxation Enhancement) T2w MRI sequences. The BBB transfer rate maps were constructed from perfusion data acquired by using a dynamic contrast-enhanced MRI technique (DCE-MRI) with Gd-DTPA bolus injection. Images acquired by a 7T research-dedicated Bruker magnet equipped with a cryogenic quadrature RF surface coil as the transmitter/receiver.

We have observed that CYP2E1 (−/−) I/R models have a lower area with abnormal BBB transfer rate in comparison to WT in I/R models. Interestingly, there was no significant damage to the BBB of CYP2E1 (−/−) I/R models as measured by the rate of leakage of the contrast agent (Gd-DTPA) from the blood into cerebral tissues through the BBB. The BBB transfer rate maps which represent a pixel-wise calculated BBB transfer rate are depicted for three consecutive slices that cover most of the ischemic lesion volume are shown in panels A and B of Figure 6. Anatomical images representing the lesion anatomy are accompanied by corresponding BBB transfer rate maps on top of panels A and B. Bar plot in panel C statistically compares the BBB transfer rate between CYP2E1(−/−) I/R and WT I/R in three slices. We observe a statistically significant (*p* < 0.01) difference between the mean of BBB transfer rate of CYP2E1(−/−) I/R and WT I/R. (Figure 6C). 

### 3.7. The 20–HETE Expression in the Brain

We measured the 20-HETE levels in WT controls and CYP2E1(−/−) mice brain with and without I/R insult. The 20-HETE level in WT controls n was 37.1 ± 2.5 ng/mL (Figure 6D). However, I/R insult triggers an increase in 20-HETE synthesis in CYP2E1(−/−) mice brain. The 20-HETE synthesis was increased significantly (*p* < 0.05) in CYP2E1(−/−) mice following the insult of I/R (52.5 ± 6.8 ng/mL, a 41% increase of WT values). Interestingly we observe that the I/R insult did not significantly increase 20-HETE synthesis in WT controls. 

## 4. Discussion 

Here, we report results from an experimental study designed to evaluate the potential of CYP2E1 inhibition as a target for neuroprotection in I/R. Our results suggest that cerebral I/R insult activates CYP2E1 and increases ROS production. Using genetic approaches, we provide evidence that oxidative enzyme CYP2E1 critically defines I/R outcome. Indeed, mice in which CYP2E1 was deleted had the best stroke outcome (Figure 1 and Figure 6). More specifically, CYP2E1(−/−) mice showed reduced GFAP and Iba-1 levels and reduced inflammatory markers following I/R (Figure 4 and Figure 5). Together, our data suggest that CYP2E1 plays a crucial role in provoking inflammation and ROS production following I/R insult. Indeed, we have developed a very interesting genetic model of ROS handling that is applied to the problem of ROS generation during focal I/R in the mouse brain. The various components of this conclusion are discussed below.

CYP2E1 is significantly expressed in different brain cell compartments, including the endoplasmic reticulum, the plasma membrane, the Golgi apparatus, as well as mitochondria [6,38,39,40,41]. The presence of CYP2E1 in these organelles highlights its role in oxidative stress and cytotoxicity in the brain. However, brain CYP2E1 expression is cell- and region-specific. For example, CYP2E1 distribution has been confirmed in the neurons of the cortex, cerebellum, and hippocampus of the human brain. In the rat brain, besides these areas, CYP2E1 is also expressed in the olfactory bulb, striatum, and thalamus [13]. CYP2E1 activation in anatomical regions is also cell-specific. For example, CYP2E1 expression has been reported in astrocytes of the cortical area one week following ischemic injury [12]. Another study using mice displayed CYP2E1 staining in glial cells and sporadic vessels throughout the hippocampus [42]. 

Various stimulants such as CNS inflammation [43], chronic ethanol treatment [44,45], as well as nicotine [26,46] induce cerebral CYP2E1 activity. The increase of CYP2E1 activity in astrocytes also has been observed following 5 min occlusion of carotid arteries of gerbil and rat model of global ischemia [12]. Notably, the authors of the same study also demonstrated an increased level of this enzyme in cortical areas following ischemic insult. In rats exposed to ethanol, brain CYP2E1 activity positively correlated with the damage to the hippocampus, cerebellum, and brain stem [44].

In this study, we confirmed that CYP2E1 protein is expressed in brain tissues of WT controls when we compare to KO mice brain tissues. Furthermore, we showed that in the MCAo mouse models, I/R insult increases CYP2E1 protein activity by (61% increase) compared to WT controls. Ischemic insult, as well as traumatic brain injury (TBI), increases the activity of phospholipase A2 (PLA2), which hydrolyzes membrane phospholipids to generate arachidonic acid (AA) [47]. An increase in AA (a substrate for CYP2E1) promotes eicosanoids (epoxyeicosatrienoic acid, EETs, and HETEs) production. Of note is that our 24 h I/R model is an acute brain injury model. However, cerebral ischemia develops over periods of hours to days after the primary event. Upstream signals, such as oxidative stress together with neutrophil and/or platelet interactions with activated endothelia, result in up-regulation of matrix metalloproteinases, plasminogen activators, and activation of endogenous adaptive and regenerative mechanisms. These detrimental factors act in orchestration with each other shortly after the ischemic injury of stroke that results in compromised integrity of the BBB and the extent of cellular swelling. The BBB is necessary to provide an optimal chemical environment for cerebral function. Protection of BBB and reduced edema by reducing the free radicals surge are correlated with improved stroke outcomes.

Free radicals surge exacerbates cerebral damage following I/R insult. However, little is known about the CYP2E1 role in free radicals production following I/R. In this study, a marked decrease was observed in cerebral I/R damage following CYP2E1 suppression. In these models, a marked reduction in lesion volume, as well as ROS activity following I/R insult, supports the notion that CYP2E1 activity plays a crucial role in oxidative stress following I/R. Of note is that these observations were not sex-specific. Although there is a sex-specific expression of some hepatic CYPs [48], there is no evidence for the brain expression.

Increased ROS activity is observed in cerebral reperfusion [49]. Considerable effort has been devoted to identifying cellular and molecular sources of excess ROS production following I/R [50]. Several studies have demonstrated multiple cellular sources of ROS production following I/R insult. For example, Kontos et al. found that endothelial cells and vascular smooth muscle cells generate ROS during reperfusion [49]. Activated microglial cells in culture have been shown to produce ROS following ischemia [51]. Authors in the same study have also shown that oligodendroglial cells are more prone to hypoxia than are astrocytes. Moreover, proinflammatory cytokines that enter the ischemic territory within 24 h can contribute to ROS formation [52]. Here, we are presenting CYP2E1 as a ROS-regulating enzyme, though our data are not specific to cerebral cell lines.

Of note is that along with CYP2E1 there are other cellular oxidase scavenger systems such as lipoxygenase (LOX) [53], cyclooxygenase (COX) [54], and NADPH oxidases (NOXs) [55]. It has been shown that the deletion of these oxidative enzymes produces a similar phenotype in protecting the vascular system from oxidative stress [54,56]. Most of the studies on the importance of cellular oxidase scavenger systems focused on using chemical inhibitions. Here, we have used a genetic approach to show the importance of CYP2E1 inhibition on reducing oxidative stress following I/R. 

Inflammation contributes significantly to the pathogenesis of post-ischemic insult [57,58,59]. Our data confirmed the activation of inflammatory markers such as IL-6, MCP-1, and TNFα following 90 min I/R at 24 h in WT mice. IL-6, MCP-1, and TNFα are major proinflammatory cytokines that upregulated following I/R insults [60,61]. However, we observed a two-fold decrease in these markers concentration in the CYP2E1(−/−) MCAo mice models of I/R compared with sham and WT controls. These proinflammatory cytokines induce the generation of ROS in non-phagocytic cells such as vascular smooth muscle cells and endothelial cells [1].

These lines of evidence indicate that CYP2E1 not only has a crucial role in oxidative stress but also contributes to proinflammatory markers activation following I/R. The connection between CYP2E1 and inflammatory markers is complicated. For example, studies have shown that IL-6 has a critical role in enabling lipopolysaccharides (LPS) to increase CYP2E1 activity by using IL-6 KO mice [62]. Therefore, positive feedback that was initiated from inflammatory markers could exacerbate CYP2E1 damaging role on I/R injury.

Here, we further explored the role of CYP2E1 activity on damaging BBB integrity following I/R insult. Our data indicate that CYP2E1 suppression alleviates BBB damage following I/R insult. To explore the underlying mechanism by which CYP2E1 suppression impacts BBB health following I/R, here we measured 20-HETE expression. Furthermore, 20-HETE is a cytochrome P450-derived metabolite of AA [63]. 20-HETE has a significant role in vascular tone regulation, CBF autoregulation, neurovascular coupling [63,64], and microvasculature function [65]. It has been shown in a rat model that in acute brain injuries following ischemia or TBI 20-HETE formation significantly increases [66]. Moreover, by using rat models of ischemia, 20-HETE has been implicated in I/R injury [15,16,18]. Our data is in line with previous observations of a surge in 20-HETE following I/R (Figure 6)**.** However, the debate remains on how a 20-HETE surge impacts I/R injury. A study connects 20-HETE vasoconstrictors property to reduced I/R injury [67]. Another study shows that 20-HETE affects vascular function through G protein-coupled receptor 75 (GPR75) [68]. We propose that a 20-HETE surge has a connection with BBB protection following I/R insult in our mouse models. Although this study demonstrates an association between CYP2E1 activity and the extent of BBB damage, additional work is required to establish causality and to explore the functional significance of CYP2E1 activity. 

In this study, histochemical experiments were designed to enable us to draw conclusions based on group statistics. On the other hand, MRI data enabled us to draw individual longitudinal analysis, as well as group analysis. We admit that employing a histological group analysis method limits the acquired knowledge of the specific CYP2E1 dynamics in each animal. Another limitation is that knocking CYP2E1 out may interfere with other detoxification mechanisms that may reduce the toxicity of ischemia. Therefore, it may confound with the goals of this study, i.e., the reduction of oxidative stress of ischemia. To avoid this confounding, we monitored animals for their health and also examined serum inflammatory markers at least twice before I/R. We planned to exclude animals if a complication happens, which was not the case in this study. As a future direction of this study, we recommend considering the conditional KO model, which is now available, to study the specific role of CYP2E1 in the brain. However, this study using CYP2E1(−/−) mice enabled us to identify whether cyp*2e1* inhibition is a crucial factor for the expression of EETs as an endothelium-derived hyperpolarizing factor (EDHF). Regarding hepatic CYP2E1 expression, it will merit investigating if CYP2E1 plays a role in liver-brain communications. Moreover, it will be interesting to investigate the morphology and 3D architecture of brain vasculature in the CYP2E1 KO mouse. 

## 5. Conclusions

In conclusion, I/R insult activates oxidative enzyme CYP2E1 that induces oxidative stress, increases inflammatory markers, and exacerbates I/R damages. Genetic inhibition of this enzyme significantly reduces oxidative stress, specifically on the BBB tight junctions following I/R. Following I/R insult, the cascade of inflammatory/oxidative stress, that CYP2E1 plays a major role, destroys the BBB tight junctions. Understanding the specific roles of the CYP on exacerbation of oxidative stress and inflammation following I/R may lead to more refined therapeutic strategies.

## Data Availability

The data presented in this study are available on request from the corresponding author. The data are not publicly available due to privacy issues.

## Supplementary Materials

The following are available online at https://www.mdpi.com/2076-3921/10/1/52/s1, Figure S1: Regional cerebral blood flow (rCBF) as measured by Laser Doppler. rCBF is measured in Contralateral and Ipsilateral of focal ischemia during occlusion and post reperfusion respectively; Figure S2: Neurological deficit following I/R insult.
