# Supplementary material for: Cytochrome P450 CYP2E1 Suppression Ameliorates Cerebral Ischemia Reperfusion Injury
Source: Antioxidants (Basel). 2021 Jan 5;10(1):52. doi: 10.3390/antiox10010052 (PMC7824747; doi:10.3390/antiox10010052)
Supplement: Supplementary file 1 [file antioxidants-10-00052-s001.pdf]

Supplementary Data

Materials and Methods:

The introduction of the C57Bl/6J background into the KO construct–In order to ensure consistency in comparing our data with other data which are mostly collected on C57BL/6 genetic background we introduced C57BL/6J genetic background into the CYP2E1 KO construct of 129/SV background. CYP2E1 KO mice were then backcrossed to C57BL/6J for at least ten generations to obtain C57BL/6J genetic background[62].

Inclusions and exclusions of the model–Successful MCAo confirmed prior to reperfusion by Laser Doppler Flowmetry (LDF; Moor Instruments, Wilmington, DE)[63]. Mice included in this study if during ischemia, LDF regional CBF dropped below 25.4 ± 1.8% of the pre-ischemic level or if post reperfusion CBF rebounded to at least 60.5 ± 3.6% of the pre-ischemic level. Mice were also excluded from this study if their body weight dropped below 30% of their pre-MCAo weight.

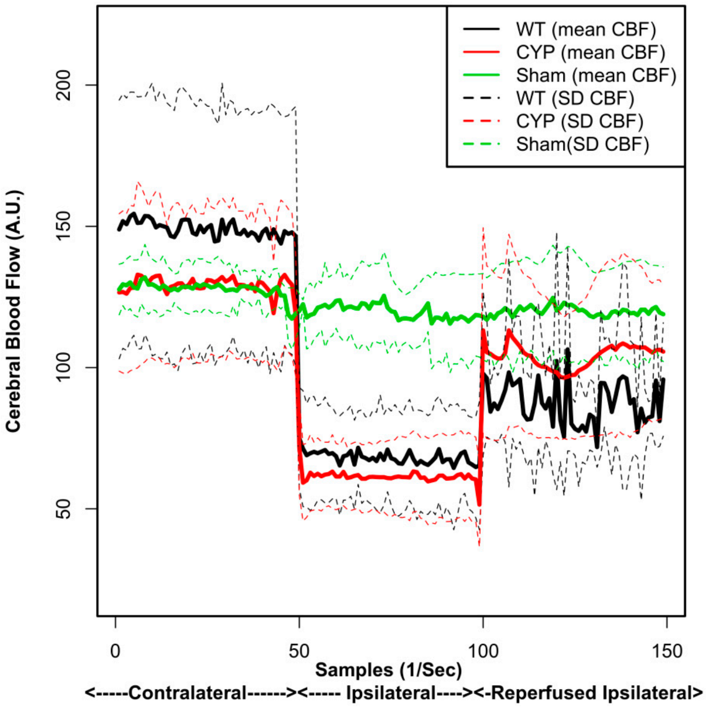

Figure S1. Regional cerebral blood flow (rCBF) as measured by Laser Doppler. rCBF is

measured in Contralateral and Ipsilateral of focal ischemia during occlusion and post reperfusion respectively. Data are represented as mean ± Standard deviation (SD). Three randomly experimental groups; Sham operated mice (n=5), CYP2E1(-/-) mice (n=7), and WT mice (n=7) were used to generate the data.

TTC staining–Slices were then incubated in a 2% solution of TTC in 0.1 M PBS (pH 7.4) at 37°C for 30 min and fixed in 10% formalin. Normal brain tissue was stained red, and the infarct tissue was white. TTC-stained brain sections were photographed using a digital camera (Powershot 400 digital camera, Canon). The infarct size was calculated by the researcher (ST) blind to the group identity, and the percentage of the infarct area with respect to the total area was digitally quantified by ImageJ. The infarct volume percentage was expressed by the sum of the infarct area of each section / the sum of the area of each section.

Preparation of brain membranes–Brains were pooled in 10 volumes of cold *homogenization buffer* (100 mM Tris (pH 7.6 at 4°C) with 0.1 mM EDTA, 0.32 M sucrose and 0.1 mM DTT) and manually homogenized on ice using a glass homogenizer. Homogenates were centrifuged twice at 3000 × g for 10 min to remove cellular and nuclear debris. Next, we took supernatants (S1) and spin (Beckman Optima TL centrifuge; TLA45 rotor) at 110,000 × g at 4°C for 100 min to yield crude cytosol (S2) and crude membrane pellet (P2). Then we resuspended pellet in homogenization buffer and spin again at ~110,000 x g to yield washed crude membrane pellet (P2′). Protein concentration was measured by BCA protein assay kit (Thermo Fisher Scientific). For immunoblotting, the resulting membrane pellets were resuspended in a storage solution containing 100 mM Tris (pH 7.4), 0.1 mM EDTA, 0.1 mM DTT, 1.15% (w/v) KCl, and 20% (v/v) glycerol, and then samples were stored in aliquots at −80°C.

CYP2E1 enzyme activity measurement–Briefly, a mixture containing 440 μL assay buffer of 100 mM potassium phosphate to PH 7.4, 10μL of 5 mM p-nitrophenol, and 25μL of 20 mM NADPH was incubated for 15 min. After that, samples (P2′, 200 μg protein) were added and incubated at 37°C for 60 min in a dark room. The reaction then terminated by adding 100μL of 20% trichloroacetic acid to the suspension. The samples were placed on ice for 2 min. After centrifugation at 10,000 × g for 5 min, 500μL of supernatant mixed with 250μL of 2 M NaOH. The absorbance of p-nitrocatechol then determined at 535nm in a multiplate reader spectrophotometer (Fluostar Optima, BMG Labtech). Activity expressed as picomoles formed per hour per micrograms of cell protein (**Figure 2**).

Immunohistochemistry protocols and antibodies for TUNEL assay–To assess the extent of brain damage, paraffin embedded coronal brain section processed for TUNEL assay. TUNEL staining was performed according to the manufacturer's instruction (Apop tag^R, S7100, Milipore Sigma USA). Briefly, the brain tissue was placed in 4% paraformaldehyde and embedded in paraffin sectioned. After washing, the sections were incubated in TDT enzyme at 37°C for one hour in a humidified chamber. The reaction was revealed with 0.06%. TUNEL signal was detected with Alexa 488-labelled secondary antibody for streptavidin. Cell nuclei were stained with DAPI. Negative controls of TUNEL staining were performed by omitting TdT. The number of apoptotic/necrotic neurons was determined by counting the NeuN/TUNEL double-stained cells in the infarct region. The percentage of TUNEL-positive cell in total DAPI positive nuclear cells was presented.

Immunohistochemistry and Western Blotting–Relative levels of Iba-1, GFAP, and β-actin in the supernatant fraction from the brain extract were determined by western blot analysis (rabbit

polyclonal antibodies: Iba-1 (ab-153696); GFAP (sc-6170), β-actin (sc-130657), Abcam Cambridge UK and Santa Cruz Biotechnology, Santa Cruz, CA), as described previously[21]. Relative intensities of western blot bands were assessed by densitometry in triplicate for each sample. Densitometric analysis was done using IQTL software (GE Life Science, Piscataway, NJ).

ROS generation measurement–Briefly, the homogenate was diluted 1:20 to obtain a concentration of 5mg tissue/ml. The reaction mixture (1ml) containing Lock's buffer (pH=7.4), 0.2ml homogenate (0.25mg protein) and 10ml of CM-H2DCFDA (5uM) was incubated for 45 min in room temperature. Samples were normalized for protein concentration. The formation of oxidized fluorescent derivative (DCF) was monitored at excitation and emission wavelengths of 488 and 525 nm, respectively, using a Multi-Mode Microplate Reader (Biotek Synergy neo2, USA) with a 20nm bandwidth. Mean intensity of each well was measured. All procedures were performed in the dark and blanks containing CM-H2DCFDA (no homogenate) were processed for measurement of autofluorescence.

Animal imaging conditions–Isoflurane gas was used for anesthesia the same way as it was used for surgery with a slightly different dosage to minimize the effect of anesthesia on CBF (induction dosage 2-2.5% and maintenance dosage 1.5%-2%; controlled for a stable respiration rate), at 1 L/min compressed air flow under spontaneous respiration throughout the entire course of each imaging session. Real time monitoring of physiological parameters (heart rate, respiratory rate, and body temperature) were conducted during each imaging session for signs of distress (Small Animal Instruments Inc., Stony Brook, NY). MR imaging protocol for each session was included sequences for anatomical and DCE-MRI.

Structural MRI– High resolution T2-weighted (T2w) anatomical images were acquired in the coronal as well as axial planes centered 5 mm caudal from the posterior edge of the olfactory bulb using a RARE (Rapid Acquisition with Relaxation Enhancement) sequence (TR/TE 4000/65 ms, field of view (FOV) 2.0 cm × 2.0 cm, slice thickness 1 mm, slice gap 0.1 mm, contiguous slices 12, matrix 256 × 128, number of averages 5, and receiver bandwidth 250 kHz). FOV of 2 cm × 2 cm and an acquisition matrix of 256 × 128 resulted in an image resolution of 0.145 × 0.296 × 1 mm^3.

DCE-MRI– Mice in the magnet received an IV injection of 0.1 mmol/kg of Gd-DTPA (MW=938 Da; Bayer Healthcare) as the contrast agent. Before the Gd-DTPA injection, a baseline T1 image was acquired. Then, Gd-DTPA was injected as a bolus into mouse tail vein via an indwelling catheter (MR compatible) followed by 15 T1 acquisitions. The following optimized MRI parameters were used to acquire each T1 data: axial plane, 2D IR-SE-EPI, TR/TE 3.24s/11.4 ms, number of IR points 16, FOV 2.0 cm x 2.0 cm, slice thickness 1.0 mm, slice gap 0.1 mm, number of slices 12, matrix 80 x 64, number of averages 4, scan time for each T1 acquisition was 2min 35sec.

Results:

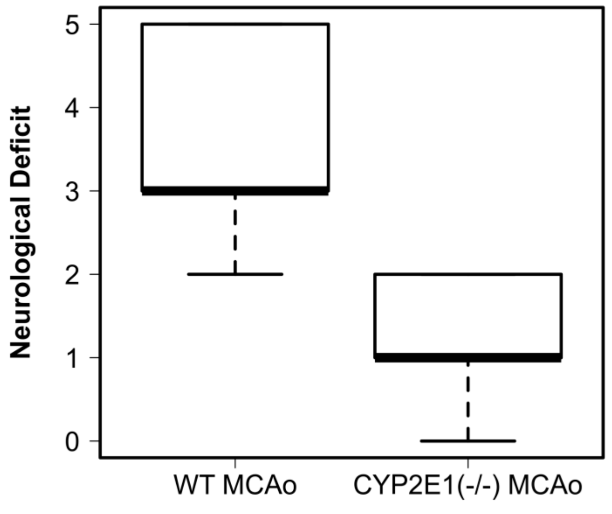

Figure S2. ***Neurological deficit following I/R insult***–The neurological deficits were assessed and scored on a 5-point scale based on the report of Longa *et al*[13]. A statistically significant differences between Wild Type (WT) MCAo group and CYP2E1 KO MCAo group was observed (n=12, $p<0.001$).
